# Capnometry successfully predicts outcome and determination of the cessation of cardiopulmonary resuscitation efforts

**DOI:** 10.1186/cc10881

**Published:** 2012-03-20

**Authors:** EH Hajdinjak, ŠG Grmec, MK Križmarić, ET Torkar, MS Skufca, DB Buić-Rerečić, MK Kovač, MZ Zelinka

**Affiliations:** 1 Center for Emergency Medicine Maribor, University of Maribor, University of Ljubljana, Slovenia, Maribor, Slovenia; 2 Community Health Centre Ljubljana, University of Ljubljana, University of Maribor, Ljubljana, Slovenia; 3 Faculty of Medicine, University of Maribor, Slovenia

## Introduction

Prognosis in patients suffering out-of-hospital cardiac arrest is poor. Higher survival rates have been observed only in patients with ventricular fibrillation who were fortunate enough to have basic and advanced life support initiated soon after cardiac arrest. An ability to predict cardiac arrest outcomes would be useful for resuscitation. Changes in expired end-tidal carbon dioxide levels during cardiopulmonary resuscitation (CPR) may be a useful, noninvasive predictor of successful resuscitation and survival from cardiac arrest, and could help in determining when to cease CPR efforts.

## Methods

This is a prospective, observational study of 1,080 cases of out-of-hospital cardiac arrest. The patients were intubated and measurements of end-tidal carbon dioxide taken. Data according to the Utstein criteria, demographic information, medical data, and partial pressure of end-tidal carbon dioxide (PetCO_2_) values were collected for each patient in cardiac arrest by the emergency physician. We hypothesized that an end-tidal carbon dioxide level of 1.9 kPa (14.3 mmHg) or more after 20 minutes and 1.8 kPa or more after 15 minutes of standard advanced cardiac life support would predict restoration of spontaneous circulation (ROSC).

## Results

PetCO_2 _after 20 minutes of advanced life support averaged 0.97 ± 0.33 kPa in patients who did not have ROSC and 4.85 ± 1.74 kPa in those who did (*P *< 0.001). PetCO_2 _after 15 minutes of advanced life support averaged 1.11 ± 0.39 kPa in patients who did not have ROSC and 3.65 ± 0.98 kPa in those who did (*P *< 0.001). End-tidal carbon dioxide values of 1.9 kPa (14.3 mmHg) or less discriminated between the 578 patients with ROSC and 502 patients without. When a 20-minute end-tidal carbon dioxide value of 1.9 kPa (14.3 mmHg) or less was used as a screening test to predict ROSC, the sensitivity, specificity, positive predictive value, and negative predictive value were all 100%. The 15-minute petCO_2 _value of 1.8 kPa had a sensitivity and NPV of 100% with high specificity PPV value (98%).

## Conclusion

End-tidal carbon dioxide levels of more than 1.9 kPa (14.3 mmHg) after 20 minutes may be used to predict ROSC with accuracy. End-tidal carbon dioxide levels should be monitored during CPR and considered a useful prognostic value for determining the outcome of resuscitative efforts and when to cease CPR in the field.

